# The Doctor Is In(ternet): The Mediating Role of Health Anxiety in the Relationship between Somatic Symptoms and Cyberchondria

**DOI:** 10.3390/jpm12091490

**Published:** 2022-09-12

**Authors:** Gianluca Santoro, Vladan Starcevic, Andrea Scalone, Josephin Cavallo, Alessandro Musetti, Adriano Schimmenti

**Affiliations:** 1 Faculty of Human and Social Sciences, UKE—Kore University of Enna, 94100 Enna, Italy; 2 Faculty of Medicine and Health, Sydney Medical School, Nepean Clinical School, Discipline of Psychiatry, University of Sydney, Sydney, NSW 2050, Australia; 3 Department of Humanities, Social Sciences and Cultural Industries, University of Parma, 43121 Parma, Italy

**Keywords:** cyberchondria, somatic symptoms, health anxiety, hypochondriasis, mediation model

## Abstract

Cyberchondria is a dysfunctional behavioral pattern characterized by an excessive and anxiety-amplifying engagement in searching for reassuring health information on the Internet. Research demonstrated that somatic symptoms and health anxiety might foster maladaptive health-related behaviors, such as cyberchondria. However, the relationships between somatic symptoms, health anxiety, and cyberchondria have been scarcely examined. Accordingly, this study aimed to test the mediating effect of health anxiety on the association between somatic symptoms and cyberchondria. Four hundred and thirty-one adults from the community (158 males, 36.66%), aged between 18 and 74, were recruited via an online survey. Participants completed self-report measures of somatic symptoms, health anxiety, and cyberchondria. A mediation analysis demonstrated that the severity of somatic symptoms predicted increased levels of cyberchondria and that health anxiety partially mediated this association. Therefore, interventions aimed at decreasing health anxiety may also play a role in decreasing the risk of developing cyberchondria.

## 1. Introduction

Cyberchondria is a dysfunctional behavioral pattern characterized by an excessive and anxiety-amplifying engagement in searching for medical or health-related information on the Internet. Cyberchondria is associated with high levels of distress and reduced perception of well-being [1,2]. Latest diagnostic and classification psychiatric systems, such as the Diagnostic and Statistical Manual of Mental Disorders, Fifth Edition Text Revision (DSM-5-TR [3]) and 11th Revision of the International Statistical Classification of Diseases (ICD-11 [4]) do not include cyberchondria among mental disorders. However, there is a growing consensus among scholars that cyberchondria is becoming a public health issue [2]. Indeed, cyberchondria has specific clinical characteristics and is associated with functional impairment. The three main features of cyberchondria are: (a) compulsive use of Internet platforms to search for medical or health-related information, usually with the purpose of obtaining reassurance about one’s own symptoms; (b) increase in the levels of distress or anxiety as a result of online health searches, with this increase persisting over time; (c) increase in online health searches and reassurance seeking over time, despite their negative consequences [5].

Cyberchondria has been considered a problematic online behavior [6]. Notably, an excessive engagement in online activities may be associated with addictive-like symptoms (e.g., withdrawal and tolerance) [7,8], which may arise during childhood or adolescence [9,10,11]. According to the compensatory model of Internet use [12], an excessive engagement in online activities may represent an attempt to cope with psychological and interpersonal difficulties [13]. For example, research has demonstrated that individuals with psychological vulnerabilities—such as insecure attachment [14,15,16], emotion dysregulation [17,18,19], maladaptive personality traits [20,21], and dissociation [22]—may excessively rely on online activities to increase their sense of belongingness or to alleviate painful feelings (e.g., health anxiety). Accordingly, the correlations between cyberchondria and problematic Internet use have been fairly strong, ranging between r = 0.43 and 0.59 [23,24,25]. Such significant association highlights relevant features that are common to both, including excessive involvement in online activities, diminished control over these activities, and continued engagement despite the negative consequences. Moreover, people with cyberchondria are at a higher risk of reporting other problematic online behaviors [26]. However, Baggio and colleagues [6] demonstrated that problematic online behaviors (including cyberchondria) occurred as distinct entities, which suggests the need to identify specific risk factors for each of these conditions.

Previous studies revealed several risk factors for cyberchondria, including health anxiety [27], obsessive-compulsive symptoms [28,29], intolerance of uncertainty [30,31,32], and negative metacognitive beliefs (i.e., the perception of uncontrollability and negative evaluation of thoughts concerning health) [33,34,35]. Notably, Zheng and colleagues [36] investigated the relevant antecedents of cyberchondria and proposed a model in which a perceived health threat arising from somatic symptoms causes health anxiety, which, in turn, leads to online health searches, whereby “information insufficiency” mediates this relationship. Online health searches have a strong and positive relationship with cyberchondria, with negative metacognitive beliefs moderating this relationship.

According to the reassurance-seeking model of cyberchondria [37], individuals with high levels of health anxiety search for health information online to find reassurance about their health concerns. Despite the failure of the previous reassurance-seeking behavior to provide sufficient relief resulting in a heightened anxiety, online health searches continue and eventually become excessive and problematic, thus constituting the behavioral pattern of cyberchondria. 

It is noteworthy that the clinical syndrome characterized by health-related worries and disease conviction has been termed “hypochondriasis” for long time [38]. However, the American Psychiatric Association [39] removed the term “hypochondriasis” from the latest versions of its classification of mental disorders, and included two different clinical syndromes that partially overlap with hypochondriasis, that is, illness anxiety disorder and somatic symptom disorder [40], which are subsumed under the category of Somatic Symptom and Related Disorders. Despite both these disorders being characterized by preoccupation for one’s own health, illness anxiety disorder is characterized by none or mild somatic symptoms, whereas somatic symptom disorder is diagnosed when an individual reports clinically relevant somatic symptoms [39]. In the ICD-11 [4], hypochondriasis is included instead among the Obsessive-Compulsive or Related Disorders. According to the ICD-11 classification, the core feature of hypochondriasis is the preoccupation or fear about the likelihood of suffering from one or more serious, progressive, or threatening-life diseases. Health worries are associated with repetitive health-related behaviors—such as, seeking evidence of illness on one’s own body, searching for information on dreaded illness and seeking reassurance—or maladaptive behaviors aimed at avoiding health information—e.g., avoiding medical appointments. 

Although the extent of overlap between hypochondriasis and cyberchondria remains unclear [2], some of the key constituents of hypochondriasis—somatic symptoms, health anxiety and reassurance seeking—are of relevance for cyberchondria, which, however, also includes the detrimental role of Internet searches in potentially reinforcing both health anxiety and reassurance-seeking behaviors. 

The relationships between somatic symptoms, health anxiety and normal and problematic help- and reassurance-seeking behaviors, such as online health searches and cyberchondria, remain insufficiently understood. Previous studies demonstrated that somatic symptoms are positively associated with health anxiety [41,42] and that somatic symptoms and health anxiety may increase the likelihood of seeking medical treatments [43,44]. Even though the severity of somatic symptoms was reported to be an independent predictor of online health searches [45], there is evidence [46] that health anxiety mediates the positive association between somatic symptoms and dysfunctional illness behaviors. 

The present study was conducted to clarify the links between somatic symptoms, health anxiety and cyberchondria. Specifically, its main aim was to examine the potentially mediating role of health anxiety in the relationship between somatic symptoms and cyberchondria in a sample of adults from the community. The following three hypotheses were tested: (a) somatic symptoms, health anxiety and cyberchondria are positively associated with each other; (b) somatic symptoms and health anxiety predict increased levels of cyberchondria; (c) the positive association between somatic symptom and cyberchondria is mediated by health anxiety.

## 2. Materials and Methods

### 2.1. Participants and Procedure

The current study involved a sample of 431 adults from the community (158 males, 36.66%), ranging in age from 18 to 74 years (M = 34.64; SD = 12.01). The average number of years of education was 16.49 (SD = 2.88). No differences between genders were found for age and years of education (see Table 1).

Participants were recruited through advertisements published on social media (e.g., WhatsApp, Facebook). All advertisements contained a link that allow people to access an anonymous online survey. People who electronically signed the informed consent were administered a sociodemographic schedule and self-report measures. All questions had to be answered to avoid missing data. Exclusion criteria were as follows: (a) age under 18 years; (b) diagnosis of a major mental disorder (e.g., schizophrenia, bipolar disorder or major depression) or intellectual disability; (c) presence of a serious medical illness (e.g., diabetes, asthma, coronary heart disease, or cancer). The study received ethical approval and was conducted in accordance with the Declaration of Helsinki.

### 2.2. Measures

A sociodemographic schedule was administered to collect information on gender, age, and years of education. The following self-report measures were used to assess the variables of interest.

The *Cyberchondria Severity Scale* (CSS [47]) is a self-report instrument which assesses cyberchondria. The short form of the CSS (CSS-12 [48,49]) was used in this study. The CSS-12 includes 12 items rated on a 5-point Likert scale (1 = “Never”; 5 = “Always”). Example of an item is “I think I am fine until I read about a serious condition online”. Total score is calculated by summing scores on all items. The CSS-12 demonstrated good psychometric properties, including good internal consistency and convergent and discriminant validity [48,49]. In the current study, Cronbach’s alpha of the CSS-12 was 0.86.

The *Whiteley Index* (WI [38,50]) is a self-report instrument that assesses symptoms of health anxiety, including health-related worries and beliefs. The WI comprises 14 dichotomous questions, whereby participants respond with “Yes” or “No” to each item. Example of an item is “Is it hard for you to believe the doctor when he tells you there is nothing for you to worry about?” Answers are coded 1 for “Yes” and 0 for “No”, except for one item which is reversely scored (i.e., “Yes” is scored 0 and “No” is scored 1). Total score is computed by summing all item scores. The WI demonstrated good test-retest reliability and validity [38]. In the current study, the KR-20 index of internal reliability for tests based on dichotomous item was 0.74.

The *Level 2*—*Somatic Symptom*—*Adult Patient* [39,51] is a self-report instrument that assesses the severity of common somatic symptoms. This instrument is adapted from the well-validated *Patient Health Questionnaire Physical Symptoms* (PHQ-15 [52]). The *Level 2*—*Somatic Symptom*—*Adult Patient* comprises a list of 15 somatic symptoms. Participants are asked to rate on a 3-point Likert scale (0 = “Not bothered at all”; 2 = “Bothered a lot”) how much each symptom has bothered them in the last seven days. For example, *Level 2*—*Somatic Symptom*—*Adult Patient* includes “chest pain” and “nausea, gas, or indigestion”. Scores on all items are summed to calculate the total score. In the current study, the Cronbach alpha of the *Level 2*—*Somatic Symptom*—*Adult Patient* was 0.81.

### 2.3. Statistical Analyses

Descriptive statistics were computed for all variables. Gender differences concerning age, years of education, somatic symptoms, health anxiety, and cyberchondria were examined through t-tests. Associations between age, years of education, somatic symptoms, health anxiety, and cyberchondria were examined through Pearson’s correlation analysis. A multiple linear regression analysis was performed to investigate the role of somatic symptoms and health anxiety as predictors of cyberchondria, taking into account the effects of sociodemographic variables (i.e., gender, age, and years of education). Finally, a mediation analysis was computed to test whether health anxiety mediated the relationship between somatic symptoms and cyberchondria. Socio-demographic variables were entered as covariates in the mediation model. The scores on the scales assessing somatic symptoms and health anxiety were mean-centered in order to reduce collinearity, and 5000 bias-corrected bootstrap samples were computed to test the significance of the indirect effect. Thus, 95% confidence intervals comprising 0 indicated a nonsignificant indirect effect. The mediation analysis was performed using Model 4 of the PROCESS Macro for SPSS [53]. A *p* value of 0.05 was set as the criterion for statistical significance.

## 3. Results

Descriptive statistics and gender differences are reported in Table 1. Females reported higher levels of somatic symptoms, health anxiety, and cyberchondria. 

Pearson’s *r* correlations are displayed in Table 2. Age was negatively associated with somatic symptoms and health anxiety, whereas years of education were negatively associated with cyberchondria. Significant and positive associations were found among somatic symptoms, health anxiety, and cyberchondria. 

Results of multiple linear regression are shown in Table 3. Somatic symptoms and health anxiety positively predicted cyberchondria. Moreover, fewer years of education were associated with higher levels of cyberchondria. 

**Figure 1:**
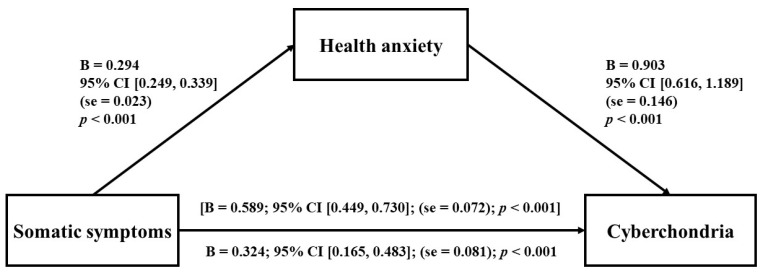
Mediating effects of health anxiety on the relationship between somatic symptoms and cyberchondria.

Mediation analysis demonstrated that the positive association between somatic symptoms and cyberchondria was partially mediated by health anxiety (Figure 1). Control for covariates showed that years of education were significantly and negatively associated with cyberchondria (B = 0.280, se = 0.117; 95% CI [−0.510, −0.501]; *p* = 0.017).

## 4. Discussion

The results of the current study supported our hypotheses, demonstrating that (a) somatic symptoms, health anxiety, and cyberchondria positively correlated with each other; (b) cyberchondria was predicted by a greater severity of somatic symptoms and higher levels of health anxiety; (c) health anxiety partially mediated the positive association between somatic symptoms and cyberchondria. 

The finding of a positive association between the severity of somatic symptoms and levels of health anxiety is in agreement with a body of research [54,55] suggesting that somatic symptoms, especially those that are medically unexplained, evoke health-related worries and beliefs which, in turn, leads to a selective attention to bodily cues. This results in a selective perception of bodily experiences, which might further increase the severity of somatic symptoms and levels of health anxiety. Indeed, studies confirm that somatic symptoms and health-related worries affect each other [42,56,57]. 

The finding of a positive association between the levels of cyberchondria and both the severity of somatic symptoms and levels of health anxiety corresponds to the findings of previous research, especially with respect to the link between cyberchondria and health anxiety [23,58,59]. The strength of the latter relationship was also confirmed by one systematic review and meta-analysis [60]. 

The multiple linear regression analysis and mediation analyses provided further insight into the relationships between somatic symptoms, health anxiety, and cyberchondria. Although a greater severity of somatic symptoms and higher levels of health anxiety both made cyberchondria more likely, health anxiety partially mediated the association between somatic symptoms and cyberchondria. This is a novel and important finding, because it indicates that the severity of somatic symptoms by itself may not be a sufficient risk factor for cyberchondria. While the severity of somatic symptoms predicts online health searches [45], the study suggests that these searches are likely to result in cyberchondria in the presence of prominent health anxiety. Similarly, Starcevic et al. [61] reported that the link between cyberchondria and somatic symptoms was indirect and weaker than the one between cyberchondria and health anxiety. Interestingly, a study by Ma et al. [46] reported that health anxiety mediated the impact of somatic symptoms on illness behavior. However, that study was conducted in patients with depression and examined functional somatic symptoms and a broad construct of illness behavior, which may also include cyberchondria. 

Gender differences were also found in the study, with females demonstrating a greater severity of somatic symptoms and higher levels of health anxiety and cyberchondria than males. These findings support previous studies suggesting that females are more prone to experiencing somatic symptoms [62,63] and internalizing symptoms, including health-related worries, and reassurance-seeking behaviors [64]. Accordingly, a recent literature review found that female gender increased the likelihood of developing cyberchondria [65].

Correlation analyses demonstrated significant associations between sociodemographic characteristics and the variables of interest. Younger age was associated with a greater severity of somatic symptoms and higher levels of health anxiety. A negative association between age and somatic symptoms is in contrast to previous studies, suggesting that older adults have a higher risk of experiencing somatic symptoms [66,67]; this calls for further research on the topic. A negative association between age and health anxiety supports previous research demonstrating that older adults experience lower levels of health anxiety [68]. Moreover, years of education negatively predicted cyberchondria. This finding is consistent with previous research and suggests that individuals with a low educational level may be less capable of evaluating the quality of online health information [69]. In this context, the lack of reassuring information could lead some less educated individuals to repeatedly search for health information on the Internet, increasing hypochondriac preoccupation and the risk for cyberchondria [70]. 

The current study comes with limitations that should be carefully addressed. Its findings are derived from an Italian community sample and may not necessarily apply to other populations, including people with severe medical conditions and those with clinically significant levels of health anxiety or cyberchondria. Although we used well-validated measures that demonstrated satisfactory psychometric properties, the self-report instruments might be subject to biases that can result in measurement error; accordingly, future research should also employ structured or semi-structured clinical interviews that provide more reliable information. Furthermore, the cross-sectional design of the study did not permit an in-depth examination of the causal relationships between variables, which calls for longitudinal studies in the future. Finally, future research might investigate whether other relevant variables (e.g., intolerance of uncertainty, health-related metacognitive beliefs, anxiety sensitivity, and problematic Internet use) mediate or moderate the relationship between somatic symptoms and cyberchondria.

## 5. Conclusions

Research is greatly needed to disentangle the specific psychopathological processes, leading some individuals suffering from somatic symptoms to excessively search for health online information, to the point that they develop cyberchondria. The present study contributes to a better understanding of cyberchondria by further elucidating the relationships between somatic symptoms, health anxiety, and cyberchondria. In particular, the study illuminates the role of somatic symptoms, which are often a starting point for online health searches. If somatic symptoms trigger massive health anxiety, these searches may be particularly likely to lead to cyberchondria because of the crucial role played by health anxiety in its development. 

Consequently, the key implication of the present study is about the prevention of cyberchondria via procedures that would reduce the risk of developing prominent health anxiety in the context of somatic symptoms. Such interventions have already been proposed [71,72,73], and now the task is to test their ability to prevent cyberchondria or at least decrease its severity.

## Figures and Tables

**Table 1:** Descriptive statistics and gender differences ^1^.

	Full Sample			Males		Females			
	(*n* = 431)			(*n* = 158)		(*n* = 273)			
	M	(SD)	Range	M	(SD)	M	(SD)	*t*_(429)_	*p*
*Age*	34.64	(12.01)	18–74	35.78	(12.20)	34.01	(11.88)	1.48	0.14
*Years of education*	16.49	(2.88)	8–21	16.15	(3.03)	16.69	(2.77)	−1.89	0.06
*Somatic symptoms*	7.59	(5.07)	0–24	5.16	(4.26)	9.00	(4.97)	−8.12	<0.01
*Health anxiety*	3.84	(2.65)	0–14	3.34	(2.50)	4.14	(2.70)	−3.06	<0.01
*Cyberchondria*	22.67	(7.58)	12–48	21.10	(7.63)	23.58	(7.40)	−3.31	<0.01

^1^ Somatic symptoms = *Level 2—Somatic Symptom—Adult Patient*, Health anxiety = *Whiteley Index* (WI), Cyberchondria = *Cyberchondria Severity Scale—Short Form* (CSS-12).

**Table 2:** Pearson’s *r* correlations among the investigated variables ^2^.

	*2.*	*3.*	*4.*	*5.*
*1. Age*	0.08	−0.17 **	−0.17 **	0.06
*2. Years of Education*	─	−0.03	−0.08	−0.11 *
*3. Somatic symptoms*		─	0.55 **	0.40 **
*4. Health anxiety*			─	0.44 **
*5. Cyberchondria*				─

^2^ Somatic symptoms = *Level 2—Somatic Symptom—Adult Patient*, Health anxiety = *Whiteley Index* (WI), Cyberchondria = *Cyberchondria Severity Scale—Short Form* (CSS-12); * *p* < 0.05, ** *p* < 0.01.

**Table 3:** Regression model predicting the severity of cyberchondria ^3^.

	B	SE	Partial *r*	*t*	*p*
*Gender*	0.68	0.72	0.05	0.95	0.34
*Age*	0.03	0.03	0.05	0.98	0.33
*Years of education*	−0.24	0.11	−0.10	−2.14	0.03
*Somatic symptoms*	0.32	0.08	0.19	4.00	<0.01
*Health anxiety*	0.90	0.15	0.29	6.19	<0.01

^3^ Gender = “male” was coded as 1 and “female” was coded as 2, Somatic symptoms = *Level 2*—*Somatic Symptom*—*Adult Patient*, Health anxiety = *Whiteley Index* (WI), Cyberchondria = *Cyberchondria Severity Scale*—*Short Form* (CSS-12); Model: F(5,425) = 27.23; *p* < 0.001; R^2^ = 0.24.

## Data Availability

The data presented in this study are available on request from the first author. The data are not publicly available due to GDPR 2016/79.

## References

[1] Starcevic V. (2017). Cyberchondria: Challenges of problematic online searches for health-related information. Psychother. Psychosom..

[2] Starcevic V., Berle D., Arnáez S. (2020). Recent insights into cyberchondria. Curr. Psychiatry Rep..

[3] American Psychiatric Association (2022). Diagnostic and Statistical Manual of Mental Disorders.

[4] World Health Organization International Mental, Behavioural or Neurodevelopmental Disorders. https://icd.who.int/browse11/l-m/en#/http%3a%2f%2fid.who.int%2ficd%2fentity%2f334423054.

[5] Vismara M., Caricasole V., Starcevic V., Cinosi E., Dell’Osso B., Martinotti G., Fineberg N.A. (2020). Is cyberchondria a new transdiagnostic digital compulsive syndrome? A systematic review of the evidence. Compr. Psychiatry.

[6] Baggio S., Starcevic V., Billieux J., King D.L., Gainsbury S.M., Eslick G.D., Berle D. (2022). Testing the spectrum hypothesis of problematic online behaviors: A network analysis approach. Addict. Behav..

[7] Griffiths M. (2005). A “components” model of addiction within a biopsychosocial framework. J. Subst. Use.

[8] Young K.S. (1998). Internet addiction: The emergence of a new clinical disorder. Cyberpsychol. Behav..

[9] Chiriţă V., Chiriţă R., Stefănescu C., Chele G., Ilinca M. (2006). Computer use and addiction in Romanian children and teenagers—An observational study. Rev. Med. Chir. Soc. Med. Nat. Iasi.

[10] Poli R., Agrimi E. (2012). Internet addiction disorder: Prevalence in an Italian student population. Nord. J. Psychiatry.

[11] Zboralski K., Orzechowska A., Talarowska M., Darmosz A., Janiak A., Janiak M., Florkowski A., Gałecki P. (2009). The prevalence of computer and Internet addiction among pupils. Postepy Hig. Med. Dosw..

[12] Kardefelt-Winther D. (2014). A conceptual and methodological critique of Internet addiction research: Towards a model of compensatory Internet use. Comput. Hum. Behav..

[13] Kardefelt-Winther D., Heeren A., Schimmenti A., van Rooij A., Maurage P., Carras M., Edman J., Blaszczynski A., Khazaal Y., Billieux J. (2017). How can we conceptualize behavioural addiction without pathologizing common behaviours?. Addiction.

[14] Musetti A., Manari T., Billieux J., Starcevic V., Schimmenti A. (2022). Problematic social networking sites use and attachment: A systematic review. Comput. Hum. Behav..

[15] Santoro G., Midolo L.R., Costanzo A., Cassarà M.S., Russo S., Musetti A., Schimmenti A. (2021). From parental bonding to problematic gaming: The mediating role of adult attachment styles. Mediterr. J. Clin. Psychol..

[16] Schimmenti A., Musetti A., Costanzo A., Terrone G., Maganuco N.R., Aglieri Rinella C., Gervasi A.M. (2021). The unfabulous four: Maladaptive personality functioning, insecure attachment, dissociative experiences, and problematic Internet use among young adults. Int. J. Ment. Health Addict..

[17] Di Blasi M., Giardina A., Giordano C., Lo Coco G., Tosto C., Billieux J., Schimmenti A. (2019). Problematic video game use as an emotional coping strategy: Evidence from a sample of MMORPG gamers. J. Behav. Addict..

[18] Liu C., Ma J.-L. (2019). Adult attachment style, emotion regulation, and social networking sites addiction. Front. Psychol..

[19] Russo A., Santoro G., Schimmenti A. (2022). Interpersonal guilt and problematic online behaviors: The mediating role of emotion dysregulation. Clin. Neuropsychiatry.

[20] Gervasi A.M., La Marca L., Costanzo A., Pace U., Guglielmucci F., Schimmenti A. (2017). Personality and Internet gaming disorder: A systematic review of recent literature. Curr. Addict. Rep..

[21] Musetti A., Mancini T., Corsano P., Santoro G., Cavallini M.C., Schimmenti A. (2019). Maladaptive personality functioning and psychopathological symptoms in problematic video game players: A person-centered approach. Front. Psychol..

[22] Guglielmucci F., Monti M., Franzoi I.G., Santoro G., Granieri A., Billieux J., Schimmenti A. (2019). Dissociation in problematic gaming: A systematic review. Curr. Addict. Rep..

[23] Fergus T.A., Spada M.M. (2017). Cyberchondria: Examining relations with problematic internet use and metacognitive beliefs. Clin. Psychol. Psychother..

[24] Durak-Batigun A., Gor N., Komurcu B., Senkal-Erturk I. (2018). Cyberchondria Scale (CS): Development, validity and reliability study. Dusunen Adam J. Psychiatry Neurol. Sci..

[25] Selvi Y., Turan S.G., Sayin A.A., Boysan M., Kandeger A. (2018). The Cyberchondria Severity Scale (CSS): Validity and reliability study of the Turkish version. Sleep Hypn..

[26] Fergus T.A., Dolan S.L. (2014). Problematic Internet use and Internet searches for medical information: The role of health anxiety. Cyberpsychol. Behav. Soc. Netw..

[27] Arsenakis S., Chatton A., Penzenstadler L., Billieux J., Berle D., Starcevic V., Viswasam K., Khazaal Y. (2021). Unveiling the relationships between cyberchondria and psychopathological symptoms. J. Psychiatr. Res..

[28] Bajcar B., Babiak J. (2021). Self-esteem and cyberchondria: The mediation effects of health anxiety and obsessive–compulsive symptoms in a community sample. Curr. Psychol..

[29] Zangoulechi Z., Yousefi Z., Keshavarz N. (2018). The role of anxiety sensitivity, intolerance of uncertainty, and obsessive-compulsive symptoms in the prediction of cyberchondria. Adv. Biosci. Clin. Med..

[30] Bajcar B., Babiak J. (2020). Neuroticism and cyberchondria: The mediating role of intolerance of uncertainty and defensive pessimism. Personal. Individ. Diff..

[31] Norr A.M., Albanese B.J., Oglesby M.E., Allan N.P., Schmidt N.B. (2015). Anxiety sensitivity and intolerance of uncertainty as potential risk factors for cyberchondria. J. Affect. Disord..

[32] Fergus T.A. (2015). Anxiety sensitivity and intolerance of uncertainty as potential risk factors for cyberchondria: A replication and extension examining dimensions of each construct. J. Affect. Disord..

[33] Airoldi S., Kolubinski D.C., Nikčević A.V., Spada M.M. (2022). The relative contribution of health cognitions and metacognitions about health anxiety to cyberchondria: A prospective study. J. Clin. Psychol..

[34] Fergus T.A., Spada M.M. (2018). Moving toward a metacognitive conceptualization of cyberchondria: Examining the contribution of metacognitive beliefs, beliefs about rituals, and stop signals. J. Anxiety Disord..

[35] Nadeem F., Malik N.I., Atta M., Ullah I., Martinotti G., Pettorruso M., Vellante F., Di Giannantonio M., De Berardis D. (2022). Relationship between health-anxiety and cyberchondria: Role of metacognitive beliefs. J. Clin. Med..

[36] Zheng H., Kyung Kim H., Joanna Sin S.-C., Theng Y.-L. (2021). A theoretical model of cyberchondria development: Antecedents and intermediate processes. Telemat. Inform..

[37] Starcevic V., Berle D. (2013). Cyberchondria: Towards a better understanding of excessive health-related internet use. Expert Rev. Neurother..

[38] Pilowsky I. (1967). Dimensions of hypochondriasis. Br. J. Psychiatry.

[39] American Psychiatric Association (2013). Diagnostic and Statistical Manual of Mental Disorders.

[40] Starcevic V. (2013). Hypochondriasis and health anxiety: Conceptual challenges. Br. J. Psychiatry.

[41] Kumar V., Avasthi A., Grover S. (2019). Correlates of worry and functional somatic symptoms in generalized anxiety disorder. Ind. Psychiatry J..

[42] Lee S., Creed F.H., Ma Y.-L., Leung C.M. (2015). Somatic symptom burden and health anxiety in the population and their correlates. J. Psychosom. Res..

[43] den Boeft M., Twisk J.W.R., Terluin B., Penninx B.W.J.H., van Marwijk H.W.J., Numans M.E., van der Wouden J.C., van der Horst H.E. (2016). The association between medically unexplained physical symptoms and health care use over two years and the influence of depressive and anxiety disorders and personality traits: A longitudinal study. BMC Health Serv. Res..

[44] Fergus T.A., Kelley L.P., Griggs J.O. (2019). The combination of health anxiety and somatic symptoms: A prospective predictor of healthcare usage in primary care. J. Behav. Med..

[45] Berle D., Starcevic V., Khazaal Y., Viswasam K., Hede V., McMullan R.D. (2020). Relationships between online health information seeking and psychopathology. Gen. Hosp. Psychiatry.

[46] Ma Y.-J., Wang D.-F., Yuan M., Long J., Chen S.-B., Wu Q.-X., Wang X.-Y., Liu T.-Q. (2019). The mediating effect of health anxiety in the relationship between functional somatic symptoms and illness behavior in chinese inpatients with depression. BMC Psychiatry.

[47] McElroy E., Shevlin M. (2014). The development and initial validation of the Cyberchondria Severity Scale (CSS). J Anxiety Disord..

[48] McElroy E., Kearney M., Touhey J., Evans J., Cooke Y., Shevlin M. (2019). The CSS-12: Development and validation of a short-form version of the Cyberchondria Severity Scale. Cyberpsychol. Behav. Soc. Netw..

[49] Soraci P., Lagattolla F., Parente G., Guaitoli E., Cimaglia R., Del Fante E., Puoti C. (2019/2020). Analisi esplorativa della Cyberchondria Severity Scale Forma Breve (CSS-12) nel contesto italiano. Mente Cura.

[50] Conti L. (2008). Repertorio Delle Scale Di Valutazione in Psichiatria.

[51] Fossati A., Borroni S., Del Corno F. (2015). Livello 2—Sintomi Somatici—Adulto.

[52] Kroenke K., Spitzer R.L., Williams J.B.W. (2002). The PHQ-15: Validity of a new measure for evaluating the severity of somatic symptoms. Psychosom. Med..

[53] Hayes A.F. (2013). Introduction to Mediation, Moderation, and Conditional Process Analysis: A Regression-Based Approach.

[54] Kellner R. (1985). Functional somatic symptoms and hypochondriasis: A survey of empirical studies. Arch. Gen. Psychiatry.

[55] Kellner R. (1987). Hypochondriasis and somatization. JAMA Netw..

[56] Groen R.N., van Gils A., Emerencia A.C., Bos E.H., Rosmalen J.G.M. (2021). Exploring temporal relationships among worrying, anxiety, and somatic symptoms. J. Psychosom. Res..

[57] Murphy K.M., McGuire A.P., Erickson T.M., Mezulis A.H. (2017). Somatic symptoms mediate the relationship between health anxiety and health-related quality of life over eight weeks. Stress Health.

[58] Fergus T.A. (2014). The Cyberchondria Severity Scale (CSS): An examination of structure and relations with health anxiety in a community sample. J. Anxiety Disord..

[59] Gibler R.C., Jastrowski Mano K.E., O’Bryan E.M., Beadel J.R., McLeish A.C. (2019). The role of pain catastrophizing in cyberchondria among emerging adults. Psychol. Health Med..

[60] McMullan R.D., Berle D., Arnáez S., Starcevic V. (2019). The Relationships between health anxiety, online health information seeking, and cyberchondria: Systematic review and meta-analysis. J. Affect. Disord..

[61] Starcevic V., Baggio S., Berle D., Khazaal Y., Viswasam K. (2019). Cyberchondria and its relationships with related constructs: A network analysis. Psychiatr. Q..

[62] Lamahewa K., Buszewicz M., Walters K., Marston L., Nazareth I. (2019). Persistent unexplained physical symptoms: A prospective longitudinal cohort study in UK primary care. Br. J. Gen. Pract..

[63] Midolo L.R., Santoro G., Ferrante E., Pellegriti P., Russo S., Costanzo A., Schimmenti A. (2020). Childhood trauma, attachment and psychopathology: A correlation network approach. Mediterr. J. Clin. Psychol..

[64] MacSwain K.L.H., Sherry S.B., Stewart S.H., Watt M.C., Hadjistavropoulos H.D., Graham A.R. (2009). Gender differences in health anxiety: An investigation of the interpersonal model of health anxiety. Personal. Individ. Diff..

[65] Vismara M., Varinelli A., Pellegrini L., Enara A., Fineberg N.A. (2022). New challenges in facing cyberchondria during the Coronavirus disease pandemic. Curr. Opin. Behav. Sci..

[66] Eslami B., Rosa M.D., Barros H., Torres-Gonzalez F., Stankunas M., Ioannidi-Kapolou E., Lindert J., Soares J.J.F., Lamura G., Melchiorre M.G. (2019). Lifetime abuse and somatic symptoms among older women and men in europe. PLoS ONE.

[67] Kocalevent R.-D., Hinz A., Brähler E. (2013). Standardization of a screening instrument (PHQ-15) for somatization syndromes in the general population. BMC Psychiatry.

[68] Gerolimatos L.A., Edelstein B.A. (2012). Predictors of health anxiety among older and young adults. Int. Psychogeriatr..

[69] Diviani N., van den Putte B., Giani S., van Weert J.C. (2015). Low health literacy and evaluation of online health information: A systematic review of the literature. J. Med. Internet Res..

[70] Starcevic V., Schimmenti A., Billieux J., Berle D. (2021). Cyberchondria in the time of the COVID-19 pandemic. Hum. Behav. Emerg. Technol..

[71] Hart J., Björgvinsson T. (2010). Health anxiety and hypochondriasis: Description and treatment issues highlighted through a case illustration. Bull. Menninger. Clin..

[72] McManus F., Surawy C., Muse K., Vazquez-Montes M., Williams J.M.G. (2012). A randomized clinical trial of mindfulness-based cognitive therapy versus unrestricted services for health anxiety (hypochondriasis). J. Consult. Clin. Psychol..

[73] Shires A., Sharpe L., Davies J.N., Newton-John T.R.O. (2020). The efficacy of mindfulness-based interventions in acute pain: A systematic review and meta-analysis. Pain.

